# Blunt dissection with a cannula prior to filler introduction may facilitate lip augmentation and enhance volume

**DOI:** 10.1016/j.jdin.2024.07.022

**Published:** 2024-08-25

**Authors:** Gisele Viana de Oliveira, Rafaela Teixeira Marques, Amanda Moreira Correa de Araujo, Marina Patrus Ananias de Souza, Doris Hexsel

**Affiliations:** a Medical Sciences School of Minas Gerais (FCMMG), Post-Graduation Department, Santa Casa de Belo Horizonte, Dermatologic Surgery Clinic, Belo Horizonte, Brazil; b Dermatologic Surgery Clinic, Santa Casa de Belo Horizonte, Belo Horizonte, Brazil; c AA Clinica de Dermatologia, Belo Horizonte, Brazil; d Hexsel Research Center, Porto Alegre, Brazil

**Keywords:** cannula, lip augmentation, filler

## Challenge

Lips are involved in interpersonal interactions and communications. Despite racial variations, full lips are considered attractive, especially when the upper lip or lower lip ratio is respected (1:1.6). Lip augmentation procedures are popular, but complications can occur.^1^^,^^2^ One of the most feared complications is vascular occlusion, which seems to be more common when needles are used for filler injections and when larger volumes are used in a single session.^2^

## Solution

**Fig 1:**
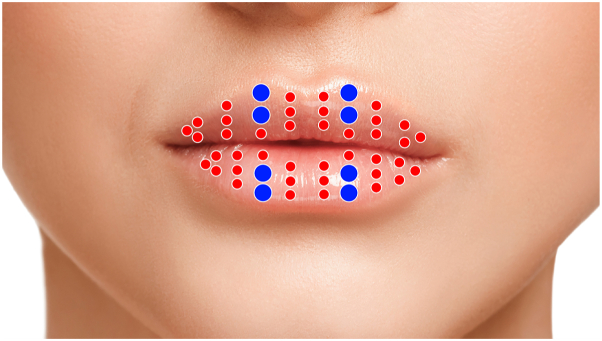
Schematic protocol with suggestions of areas where filler should be deposited, with quantities (considering a total of 1 mL). Upper portion of the lip—total volume: 0.4 mL. *Red* dots indicate a lower volume (0.015 mL), whereas *blue* dots, a double quantity (0.03 mL). Lower portion of the lip—total volume: 0.6 mL. *Red* dots indicate lower volume (0.02 mL), whereas *blue* dots, a double quantity. This allows for larger quantities on the tubercles and preserves the upper to lower lip proportion.

**Fig 2:**
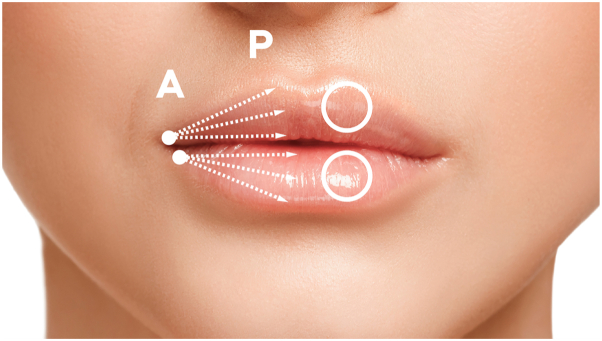
**A**, oral commissure, where the anesthetic is placed, correspondent to the cannula entrance point; **P,** philtrum; *arrows* show the blunt dissection trajectory (upper and lower portion of the lip); lateral tubercles are marked with *circles*.

We start the procedure by performing a delicate submucosal blunt dissection of the first half of the whole superior aspect of the lip, using a cannula. Only after this first step, the cannula is introduced to deposit filler in the lip contour, followed by subsequent steps until reaching the wet lip. Video 1 (available on www.jaad.org) shows that after anesthesia, the cannula is introduced in the oral commissure and the submucosal dissection takes place (Video 1, Fig 1). The cannula is then directed to the philtrum in the previously dissected area, leaving the filler during the withdraw in 3 to 4 subsequent columns. The same procedure is repeated in the other half of the upper lip, followed by the lower lip (Fig 2). This previous dissection creates a space, facilitates the diffusion of the filler throughout the whole area, enhances the impression of extra volume, and possibly decreases the risks of vascular occlusion. Table I shows tips and guidelines to achieve better outcomes after a lip augmentation procedure.

**Table I:** Tips and tricks to improve safety during injection

The blunt dissection previous to the injection of filler detaches fibrotic subdermal areas, requiring for lower filler amounts to volumize the lip.
Stay between the transition between dry and wet vermillion, considered safer area than wet area.
24-22 Gauge cannulas are preferred; cannulas with diameter <25 Gauge might pose extra risks of vascular occlusion.
Inject very slowly and do not use the product to achieve dissection; instead, inject during cannula retraction and after complete dissection.
Prefer delicate procedures and gradual changes-1 syringe per session, if patient desires for greater volumes, schedule several sessions with at least 1-mo intervals.
Keep an anatomical protocol: respect the ratio between upper and lower lip
Project upper and lower lip tubercles. Instead of injecting in the mid area, very close to the wet vermillion, project the cannula tip to the dry lip surface; it will probably decrease risks of arterial injection.
Treat surrounding areas before injecting lips, it may help achieving a better outcome.

## Conflicts of interest

Dr Oliveira receives Medical Equipment of Vydence and Compensation in scar course Scartech, but has no conflict of interest regarding this study. Dr Hexsel is a paid consultant for Allergan, Galderma and Merz, but has no conflicts of interest for this study. Drs Marques, Araujo and Souza have no conflicts of interests to declare.
